# Antineuropathic effect of 7-hydroxy-3,4-dihydrocadalin in streptozotocin-induced diabetic rodents

**DOI:** 10.1186/1472-6882-14-129

**Published:** 2014-04-07

**Authors:** Héctor Isaac Rocha-González, Magali Ramírez-Aguilar, Vinicio Granados-Soto, Juan Gerardo Reyes-García, Jorge Elías Torres-López, Juan Carlos Huerta-Cruz, Andrés Navarrete

**Affiliations:** 1 Sección de Estudios de Posgrado e Investigación, Escuela Superior de Medicina, Instituto Politécnico Nacional, Plan de San Luis y Díaz Mirón s/n, México, D.F. 11340, Mexico; 2 Facultad de Química, Departamento de Farmacia, Universidad Nacional Autónoma de México, México, D.F. 04510, Mexico; 3 Departamento de Farmacobiología, Cinvestav, Sede Sur, México, D.F, Mexico; 4 División Académica de Ciencias de la Salud, Laboratorio Mecanismos del Dolor, Centro de Investigación, Universidad Juárez Autónoma de Tabasco, 86150 Villahermosa, Tabasco, Mexico; 5 Hospital Regional de Alta Especialidad “Dr. Juan Graham Casasús”, 86103 Villahermosa, Tabasco, Mexico

**Keywords:** 7-Hydroxy-3,4-dihydrocadalin, Allodynia, Hyperalgesia, Motor activity, Neuropathic pain, Oxidative stress, Pregabalin

## Abstract

**Background:**

Painful neuropathy is the most common and debilitating complication of diabetes and results in hyperalgesia and allodynia. Hyperglycemia clearly plays a key role in the development and progression of diabetic neuropathy. Current therapeutic approaches are only partially successful and they are only thought to reduce the pain associated with peripheral neuropathy. Some natural products offer combined antioxidant, anti-inflammatory and antinociceptive properties that may help to treat in a more integrative manner this condition. In this regard, the purpose of this study was to investigate the antineuropathic effect of 7-hydroxy-3,4-dihydrocadalin in streptozotocin-induced diabetic rats and mice without glucose control as well as the possible mechanism of action involved in this effect.

**Methods:**

Rats and mice were injected with 50 or 200 mg/kg streptozotocin, respectively, to produce hyperglycemia. The formalin test and von Frey filaments were used to assess the nociceptive activity. Rota-rod was utilized to measure motor activity and malondialdehyde assay to determine anti-oxidative properties.

**Results:**

After 3 weeks of diabetes induction, chemical hyperalgesia was observed in streptozotocin-injected rats. Oral acute administration of 7-hydroxy-3,4-dihydrocadalin (0.3–30 mg/kg) decreased in a dose-dependent manner formalin-evoked hyperalgesia in diabetic rats. In addition, methiothepin (non-selective 5-HT receptor antagonist, 1 mg/kg, i.p.) and ODQ (guanylyl cyclase inhibitor, 2 mg/kg, i.p.), but not naltrexone (opioid receptor antagonist, 1 mg/kg, s.c.), prevented 7-hydroxy-3,4-dihydrocadalin-induced antihyperalgesic effect. The anti-hyperalgesic effect of 7-hydroxy-3,4-dihydrocadalin was similar to that produced by pregabalin (10 mg/kg, p.o.). Furthermore, oral acute administration of 7-hydroxy-3,4-dihydrocadalin (30 mg/kg) reduced streptozotocin-induced changes in malondialdehyde concentration from plasma samples. Unlike pregabalin, 7-hydroxy-3,4-dihydrocadalin did not affect motor activity. Six weeks after diabetes induction, tactile allodynia was observed in the streptozotocin-injected rats. At this time, oral administration of 7-hydroxy-3,4-dihydrocadalin (30 mg/kg) or pregabalin (10 mg/kg) reduced in a similar way tactile allodynia in diabetic rats. Finally, chronic oral administration of 7-hydroxy-3,4-dihydrocadalin (30-300 mg/kg, 3 times/week, during 6 weeks), significantly prevented the development of mechanical hyperalgesia and allodynia in streptozotocin-induced diabetic mice.

**Conclusions:**

Data suggests that 7-hydroxy-3,4-dihydrocadalin has acute and chronic effects in painful diabetic neuropathy. This effect seems to involve antioxidant properties as well as activation of 5-HT receptors and inhibition of guanylyl cyclase enzyme.

## Background

Diabetes mellitus is one of the most common chronic medical conditions affecting over 366 million people worldwide, causing 4.6 million deaths each year [1]. Diabetes leads to several complications including retinopathy, nephropathy and neuropathy. Diabetic neuropathy is the most common and debilitating complication of diabetes and results in pain, decreased motility, and leg amputation. The prevalence of neuropathy is estimated to be about 8% in newly diagnosed diabetic patients and greater than 50% in patients with long-standing disease [2,3]. Pain associated with diabetes can occur either spontaneously or as a result of exposure to only mildly painful stimuli (hyperalgesia) or to stimuli not normally perceived as painful (allodynia) [4,5].

Diabetes occurs when the pancreas does not produce enough insulin, or when the body cannot effectively use the insulin produced. This leads to an increased concentration of glucose in the blood. Hyperglycemia clearly plays a key role in the development and progression of diabetic neuropathy as it activates secondary glucose metabolic pathways, such as the polyol and hexosamine pathways; protein kinase C isoforms and advanced glycation end products. Collectively, these pathways cause an imbalance in the mitochondrial redox state of the cell leading to formation of reactive oxygen species that in turn promote expression of genes involved in inflammation and neuronal dysfunction, for review see [3,6]. Although, considerable progress has been made toward understanding the mechanisms leading to diabetic neuropathy, the etiology of this condition is not fully understood yet.

Current accepted therapeutic approaches are only partially successful as they only decrease pain intensity associated to this condition; however, they do not eliminate the root causes. In this regard, anticonvulsants, tricyclic antidepressants and opioids have become the mainstay in the treatment of diabetic neuropathic pain [7,8]. However, these drugs have a limited effect or may cause intolerable side-effects. Therefore, other options of treatment are needed. An alternative is natural product-derived compounds which represent a great structural diversity. Some of these may offer combined antioxidant, anti-inflammatory and antiallodynic properties, as well as novel targets to treat neuropathic pain. Scientific evidence shows that the antineuropathic effect of most of natural products can be mainly explained by activation of opioid, serotonergic and nitrergic systems [9-13], and by antioxidant properties [14-16].

*Heterotheca inuloides Cass* (Asteraceae) or Mexican arnica is a medicinal plant widely used in the Mexican folk medicine to treat several painful conditions such as wounds, bruises, rheumatisms, colic and other conditions associated to visceral and somatic inflammatory process [17]. Recent evidence obtained in our laboratory points out that 7-hydroxy-3,4-dihydrocadalin seems to be one of the main sesquiterpenoid responsible of the antinociceptive effect of this plant in inflammatory conditions [18]. Notwithstanding, considering that 7-hydroxy-3,4-dihydrocadalin has antioxidant [19-21] and anti-inflammatory [18,22] properties, the most important therapeutic effect of 7-hydroxy-3,4-dihydrocadalin could be found it in the treatment of chronic pain, specially, in those painful conditions where there is an imbalance of oxidative stress and systemic inflammatory state as painful diabetic neuropathy.

Based on the above considerations, this work was undertaken to investigate the effects of 7-hydroxy-3,4-dihydrocadalin in streptozotocin-induced diabetic rats and mice without blood glucose control as well as the possible participation of opioid, serotonergic, nitrergic and antioxidant systems in the generation of this effect.

## Methods

### Animals

All experiments on acute treatment of 7-hydroxy-3,4-dihydrocadalin were performed in adult male Wistar rats weighting between 220 and 250 g, whereas experiments on chronic treatment of 7-hydroxy-3,4-dihydrocadalin were carried out in male ICR mice ranging between 28 and 30 g. Animals were obtained from Centro UNAM-Harlan (Harlan México, S.A. de C.V.) and they were fasted for 12-h before oral drug administration. All experiments are in compliance with the requirements published by SAGARPA in the Technical Specifications for the Production, Care and Use of Laboratory Animals (NOM-062-ZOO-1999), Guidelines National Institute of Health Guide for the Care and Use of Laboratory Animals (NIH Publications No. 80-23, revised 1996) and Guidelines on Ethical Standards for Investigation of Experimental Pain in Animals [23]. In addition, the experiments were approved by Institutional Committee for the Care and Use of Laboratory Animals from Faculty of Chemistry (Protocol number FQ/CICUAL/021/11). Every effort was done to minimize pain and suffering in animals and the number of rats used was the minimal required to obtain significant statistical power.

### Plant material

Dried flowers of *Heterotheca inuloides* were kindly provided by Laboratorios Mixim S.A. de C.V. (Mexico City) from its own growing fields in August 2010. Biologist and M.Sc. Abigail Aguilar performed a formal identification of the plant material and a voucher specimen was deposited at the herbarium of the 21^st^ Century National Medical Center (Mexico City), with the number 15972.

### Extraction of 7-hydroxy-3,4-dihydrocadalin

**Figure 1:**
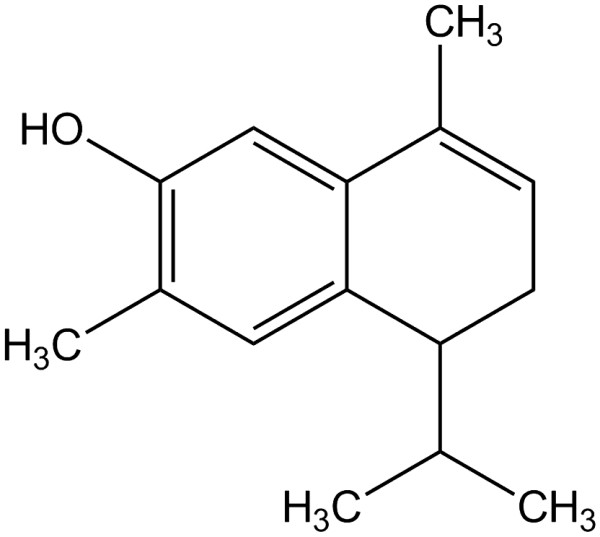
Chemical structure of 7-hydroxy-3,4-dihydrocadalin.

7-Hydroxy-3,4-dihydrocadalin was chosen as a candidate for treatment of painful diabetic neuropathy from a bioassay guided fractionation previous study, on the basis it was the main metabolite isolated from *Heterotheca inuloides* with antinociceptive activity in inflammatory pain models and as it combines antinociceptive, antioxidative and anti-inflammatory properties. 7-Hydroxy-3,4-dihydrocadalin was extracted from air-dried ground inflorescences of *Heterotheca inuloides* (1500 g) as was previously described by our laboratory group [18]. Briefly, inflorescences were extracted three times for three days each with 20 L of hexane by maceration at room temperature (~ 22°C). After evaporation of solvent under vaccum, 25 g hexane extract (1.67%) were obtained. Then, 7-hydroxy-3,4-dihydrocadalin was separated in a silica chromatography column with hexane and purified by recrystallization in hexane at 4°C. Purity (99.8%) of 7-hydroxy-3,4-dihydrocadalin was confirmed by melting point (103-104°C) [24] and Agilent 6890 N gas chromatograph with an automatic liquid sampler Agilent 7683B coupled to a LECO Pegasus 4D mass spectrometer (LECO, St. Joseph, MI, USA). Chemical structure of 7-hydroxy-3,4-dihydrocadalin (Figure 1) was confirmed by comparison of ^1^H- and ^13^C-nuclear magnetic resonances data with those previously reported [25].

### Drugs

7-Hydroxy-3,4-dihydrocadalin was prepared in deionized water with 10% Tween 80. Methiothepin (1-[10,11 dihydro-8-(methylthio) dibenzo [b,f] thiepin-10-yl]-4-methylpiperazine mesylate salt), pregabalin ((S)-3-(aminomethyl)-5-methylhexanoic acid) and naltrexone (naltrexone hydrochloride) were dissolved in 0.9% saline solution, while ODQ (1H-[1,2,4] oxadiazolo[4,3-a]quinoxalin-1-one) was prepared in 0.9% saline solution with 20% dimethylsulfoxide. Streptozotocin (N-(methylnitrosocarbamoyl)-α-D-glucosamine) was dissolved in deionized water. All drugs were freshly prepared before each experiment and were purchased from Sigma-Aldrich (St. Louis, MO, USA), with exception of 7-hydroxy-3,4-dihydrocadalin, which was extracted from dried flowers of *Heterotheca inuloides*.

### Induction of experimental diabetes by streptozotocin

Rats or mice were intraperitoneally injected with 50 or 200 mg/kg of streptozotocin, respectively, to produce experimental diabetes [26,27]. Control animals (weight-matched) received saline 0.9%. Diabetes was confirmed one day after streptozotocin injection and 12 h before acute experiments by measurement of tail vein blood glucose levels with a glucometer OneTouch® UltraMini™ (LifeScan Inc., Milpitas, CA, USA). Only rats with a blood glucose level ≥ 350 mg/dL were included in the study. For chronic experiments in mice, glucose levels were monitored each week during six weeks and all mice maintained more than 350 mg/dL of glucose throughout the experiment.

### Measurement of hyperalgesia in rats

To determine the antihyperalgesic role of 7-hydroxy-3,4-dihydrocadalin, the formalin test was performed as described elsewhere [28]. Streptozotocin-induced diabetic rats (after 3 weeks) were placed in open observation chambers for 30 min to allow them to acclimate to their surroundings; then they were removed and gently restrained while the dorsum of the hind paw was injected with 50 μL of 0.5% formalin using a 30-gauge needle. The animals were returned to the chambers and the nociceptive behavior was observed immediately after formalin injection. Mirrors were placed in each chamber to enable unhindered observation. Nociceptive behavior was quantified as the number of flinches of the injected paw during 1-min periods every 5 min, up to 60 min after formalin injection [29]. Flinching was readily discriminated and was characterized as rapid and brief withdrawal, or as flexing of the injected paw. At the end of the experiments, rats were sacrificed in a CO_2_ chamber.

### Measurement of allodynia in rats

Tactile allodynia was tested in rats with 6 weeks of streptozotocin-induced experimental diabetes as previously described [30]. Briefly, rats were transferred to a clear plastic, wire mesh-bottomed cages and allowed to acclimatize for 30 min. von Frey filaments (Stoelting, WoodDale, IL, USA) were used to determine the 50% paw withdrawal threshold using the up–down method [31]. A series of filaments, starting with one that had a buckling weight of 2 g, were applied in consecutive sequence to the plantar surface of the right hind paw with a pressure causing the filament to buckle during 5 seconds. Lifting of the paw before of 5 seconds indicated a positive response and prompted the use of the next weaker filament whereas that absence of a paw withdrawal after 5 seconds indicated a negative response and prompted the use of the next filament of increasing weight. This paradigm continued until four more measurements had been made after the initial change of the behavioral response or until 5 consecutive negative (assigned a score of 15 g) or four consecutive positive (assigned a score of 0.25 g) responses had occurred. The resulting scores were used to calculate 50% of withdrawal threshold, by using the formula:

$$50\%\;g\;\mathrm{threshold}\;=\;\frac{10^{\left(X_{f}\;+\;\kappa \delta \right)}}{10\;000}$$

where Xf = the value (in log units) of the final von Frey filament used, κ = the value from table for the pattern of positive and negative responses published previously [32], and ∂ = the mean difference (in log units) between stimuli. 50% threshold withdrawal was assessed before drug administration and every 60 min during 8 h and at 24 h after drug administration. Allodynia was considered to be present when paw withdrawal threshold was below 4 g as was described above [32]. Diabetic rats with a basal withdrawal threshold above 4 g were not included for the experiments.

### Measurement of hyperalgesia and allodynia in mice

Secondary mechanical allodynia and hyperalgesia in streptozotocin-induced diabetic mice were assessed by a modified method described above [33,34]. Mice were placed in testing cages with a wire mesh bottom and allowed to acclimate for 30 min. Baseline measurements were recorded first. Two von Frey filaments (Stoelting Co, Wood Dale, IL, USA; bending forces of 0.008 g [1.65] and 1 g [4.08]) were applied 10 times in each testing set at the base of the third toe on the plantar surface on right hindpaw. Three trials were completed to get an average of the number of paw withdrawal responses. Under normal conditions, a force of 0.008 g does not activate cutaneous nociceptors, and therefore, it does not cause paw withdrawal in normal mice. Accordingly, the occurrence of responses in diabetic mice indicates allodynia. On the other hand, a force of 1 g or more is considered a noxious stimulus, and hyperalgesia occurs when there is an increased response to this stimulus.

### Measurement of malondialdehyde

Determination of malondialdehyde concentration in streptozotocin-induced diabetic rat plasma (3 weeks) was carried out according to the manufacturer´s instructions (Cayman Chemical Company, Ann Arbor, MI, USA). Concisely, 3 mL of blood were obtained from femoral vein of anesthetized rats in heparinized tubes; blood was centrifuged at 1000 × g for 10 min at 4°C. Then, plasma (100 μL) was mixed with SDS (100 μL) and 4 mL of color reagent in 5-mL tubes. Mixture was boiled during 1 h and placed in an ice bath for 10 min to stop reaction. After that, tubes were centrifuged at 1 600 × g during 10 min at 4°C and read at 540 nm. The calibration curve was linear from 5 to 400 μM malondialdehyde (r^2^ = 0.998).

### Evaluation of motor activity

Motor activity was assessed using a rota-rod apparatus for rats (Panlab 8500, Cornellá, BCN, Spain) as was described above [35]. Diabetic rats (3 weeks) were trained for 2 days by walking them each day on an accelerating rota-rod (from 4 to 40 r.p.m. in 10 min). On the test day, trained animals were tested 1 h after drug administration (time of peak drug effect). Motor performance was considered as the latency to fall off the rota-rod apparatus determined from the mean time in three trials for each rat at each time.

### Study design

Independent groups of animals (n = 5-7) were used for each experimental condition. In order to define the best time to administer 7-hydroxy-3,4-dihydrocadalin, diabetic rats (3 weeks) received an oral administration of vehicle (saline solution with 10% tween 80) or 7-hydroxy-3,4-dihydrocadalin (30 mg/kg, p.o.) at 30 and 60 min before formalin injection (50 μL). These times were selected from pilot experiments in our laboratory. Since the best antinociceptive effect was observed at the 60 min pre-treatment (data not shown), dose–response curve to assess the acute antihyperalgesic effect of 7-hydroxy-3,4-dihydrocadalin was carried out giving vehicle or increasing doses of 7-hydroxy-3,4-dihydrocadalin (0.03 – 30 mg/kg) or a single dose (10 mg/kg) of pregabalin (positive control) 60 min before subcutaneous injection (50 μL) of 1% formalin into the dorsal surface of the right hindpaw. In order to establish the potential participation of guanylyl cyclase enzyme or serotonin and opioid receptors on 7-hydroxy-3,4-dihydrocadalin-induced systemic antihyperalgesic effect, ODQ (2 mg/kg), methiothepin (1 mg/kg) or naltrexone (1 mg/kg) were administered 30 min after a single dose of 7-hydroxy-3,4-dihydrocadalin (30 mg/kg) and 30 min before 1% formalin injection. To corroborate the lack of effect of ODQ, methiothepin and naltrexone by themselves at the tested dose with 7-hydroxy-3,4-dihydrocadalin, they were administered alone 30 min before 1% formalin injection into the right paw.

In an attempt to determine the antioxidant properties of 7-hydroxy-3,4-dihydrocadalin in streptozotocin-induced diabetic rats, the malondialdehyde concentration was measured in plasma samples of naïve and diabetic (3 weeks) rats treated with vehicle or 7-hydroxy-3,4-dihydrocadalin (30 mg/kg) 60 min before taking a blood sample from the femoral vein. In order to evaluate the effect of 7-hydroxy-3,4-dihydrocadalin (30 mg/kg) and pregabalin (10 mg/kg) on motor activity, diabetic rats (3 weeks) were orally administered with vehicle (saline solution with 10% tween 80) or a single dose of 7-hydroxy-3,4-dihydrocadalin or pregabalin 60 min before to measure the fall latency time in a rota-rod apparatus.

To confirm the acute antineuropathic effect of 7-hydroxy-3,4-dihydrocadalin, naïve and streptozotocin-induced diabetic rats of 6 weeks were administered with vehicle or the highest dose tested of 7-hydroxy-3,4-dihydrocadalin (30 mg/kg) or pregabalin (10 mg/kg) in the formalin test. After oral administration, antiallodynic effect of treatments was evaluated by von Frey filaments measuring 50% paw mechanical withdrawal threshold during 24 h [31]. Finally, in order to establish whether 7-hydroxy-3,4-dihydrocadalin was able to delay the development of streptozotocin-induced diabetic neuropathy, naïve or diabetic mice were orally treated with vehicle or increasing doses of 7-hydroxy-3,4-dihydrocadalin (30 – 300 mg/kg) on Mondays, Wednesdays and Fridays of each week during 6 weeks. In this paradigm, hyperalgesia and allodynia were measured all Mondays before drug administration as the number of hind paw withdrawal responses at 0.008 g and 1 g, respectively.

The scheduling of doses and drug administration was based on previously reported studies [36,37] and on pilot studies in our models. The observer was unaware of the treatment given to each animal. Rats in all groups were tested for possible side effects such as reduction of righting, stepping, and corneal and pinna reflexes, before and after drug treatment, as proposed elsewhere [38].

### Data analysis and statistics

Results of malondialdehyde concentration and motor activity are presented in bar plots as means ± S.E.M. for at least five animals per group, whereas bar graphs of nociceptive experiments were made of the area under the curve from time courses by the trapezoidal rule as was previously described [39]. Time courses were constructed by plotting the number of flinches (hyperalgesia in rats) or paw withdrawal threshold in grams (allodynia in rats) or the number of paw withdrawal responses (hyperalgesia and allodynia in mice) as a function of time. All nociception results are presented as means ± S.E.M. for 5-7 animals per group. One-way analysis of variance (ANOVA) followed by the Tukey’s test was used to compare differences between treatments. In addition, two-way ANOVA followed by the Bonferroni´s test was carried out to compare differences between treatments in a time course. Differences were considered statistically significant when *P* < 0.05.

## Results

**Figure 2:**
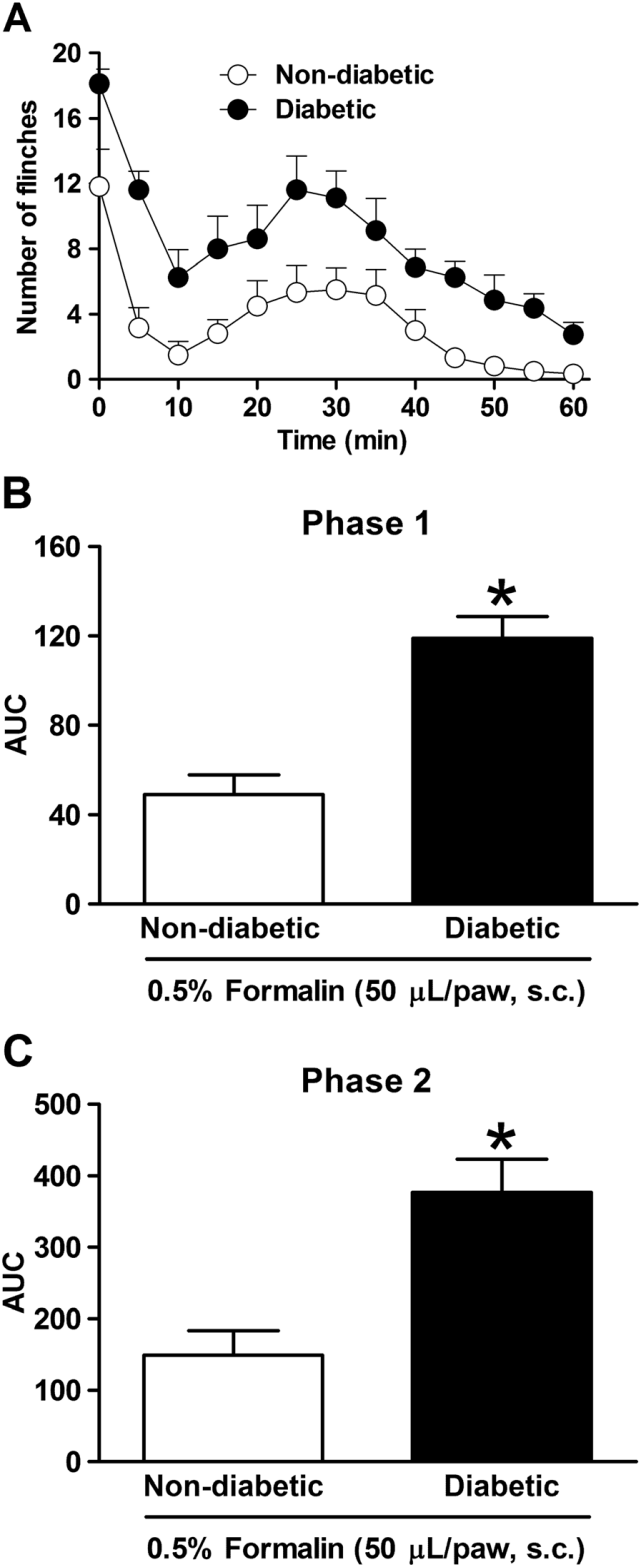
**Flinching behavior induced by subcutaneous (s.c.) injection of 0.5% formalin to non-diabetic and diabetic rats.** Time courses **(A)** and bar plots show formalin-induced hyperalgesic effect during phase 1 **(B)** and phase 2 **(C)** in diabetic rats administered with 60 mg/kg (i.p.) of streptozotocin 3 weeks before. Data are expressed as the mean ± E.E.M. of seven animals per group. *Significantly different (*P* < 0.05) from non-diabetic group, as determined by Student’s test. AUC: area under the curve.

Formalin-evoked flinching behavior in streptozotocin-induced diabetic rats Streptozotocin, but not distilled water, injection in non-fasted rats caused hyperglycemia. The blood glucose level measured in these rats was 116.6 ± 2.1 mg/dL and 112.5 ± 3.0 mg/dL before distilled water or streptozotocin injection, and 116.4 ± 1.7 mg/dL and 559.5 ± 9.6 mg/dL 3 weeks after distilled water or streptozotocin injection, respectively. Furthermore, diabetic rats had an increase in food and water intake, showed polyuria and did not gain body weight (data not shown). Both the non-diabetic and diabetic (3 weeks) groups exposed to subcutaneous injection of 0.5% formalin exhibited a biphasic pattern of flinching. Phase 1 of the nociceptive response began immediately after formalin administration and then declined gradually in approximately 10 min. Phase 2 began about 15 min after formalin administration and lasted about 1 h, as previously reported [29]. Injection of 0.5% formalin into the right paw displayed a greater flinching behavior in diabetic compared to non-diabetic rats (Figure 2A). Actually, analysis of formalin-evoked nociceptive behavior as area under curve (AUC) showed that increment in the flinching frequency of diabetic rats was statistically different (*P* < 0.05) compared to non-diabetic rats in both phases of the test (Figure 2B,C).

### Antihyperalgesic effect of acute administration of 7-hydroxy-3,4-dihydrocadalin in diabetic rats

**Figure 3:**
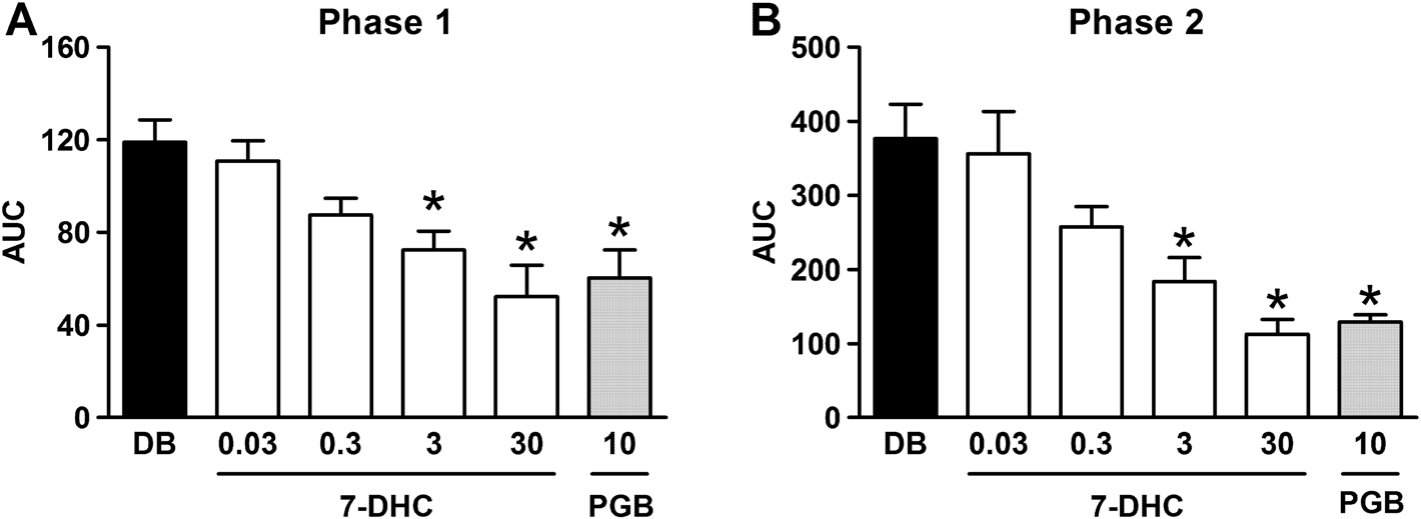
**Antihyperalgesic effect induced by oral administration of increasing doses of 7-hydroxy-3,4-dihydrocadalin (7-DHC) or 10 mg/kg pregabalin (PGB) in diabetic rats (3 weeks) during phase 1 (A) and phase 2 (B) of the 0.5% formalin test. **7-DHC and PGB were given 1 h before formalin injection. Data are expressed as the mean ± E.E.M. of six animals per experimental group. *Significantly different (*P* < 0.05) from the control group (diabetic), as determined by analysis of variance followed by Tukey´s test. AUC: Area under the curve.

Oral administration of 7-hydroxy-3,4-dihydrocadalin (30 mg/kg) reduced formalin-induced flinching behavior at 30 and 60 min pre-treatment. However, the best antinociceptive effect was observed at 60 min pre-treatment (data not shown) and this pre-treatment time was used in the following experiments. Under this condition, 7-hydroxy-3,4-dihydrocadalin decreased in a dose-dependent manner and significantly (*P* < 0.05) the number of flinches during both phases of the formalin test in diabetic rats (Figure 3A,B). In addition, 7-hydroxy-3,4-dihydrocadalin (30 mg/kg) showed to be equi-effective in percentage of antinociception to pregabalin (10 mg/kg) during phase 1 (55.9 ± 11.4% and 49.1 ± 9.9%, respectively) and phase 2 (70.0 ± 5.2% and 65.7 ± 2.7%, respectively) of the formalin test. Higher doses of pregabalin were not compared with 7-hydroxy-3,4-dihydrocadalin due to the fact that pregabalin showed evident motor side-effects that affected flinching behavior.

### Effect of methiothepin, naltrexone and ODQ on the antihyperalgesic effect of 7-hydroxy-3,4-dihydrocadalin in diabetic rats

**Figure 4:**
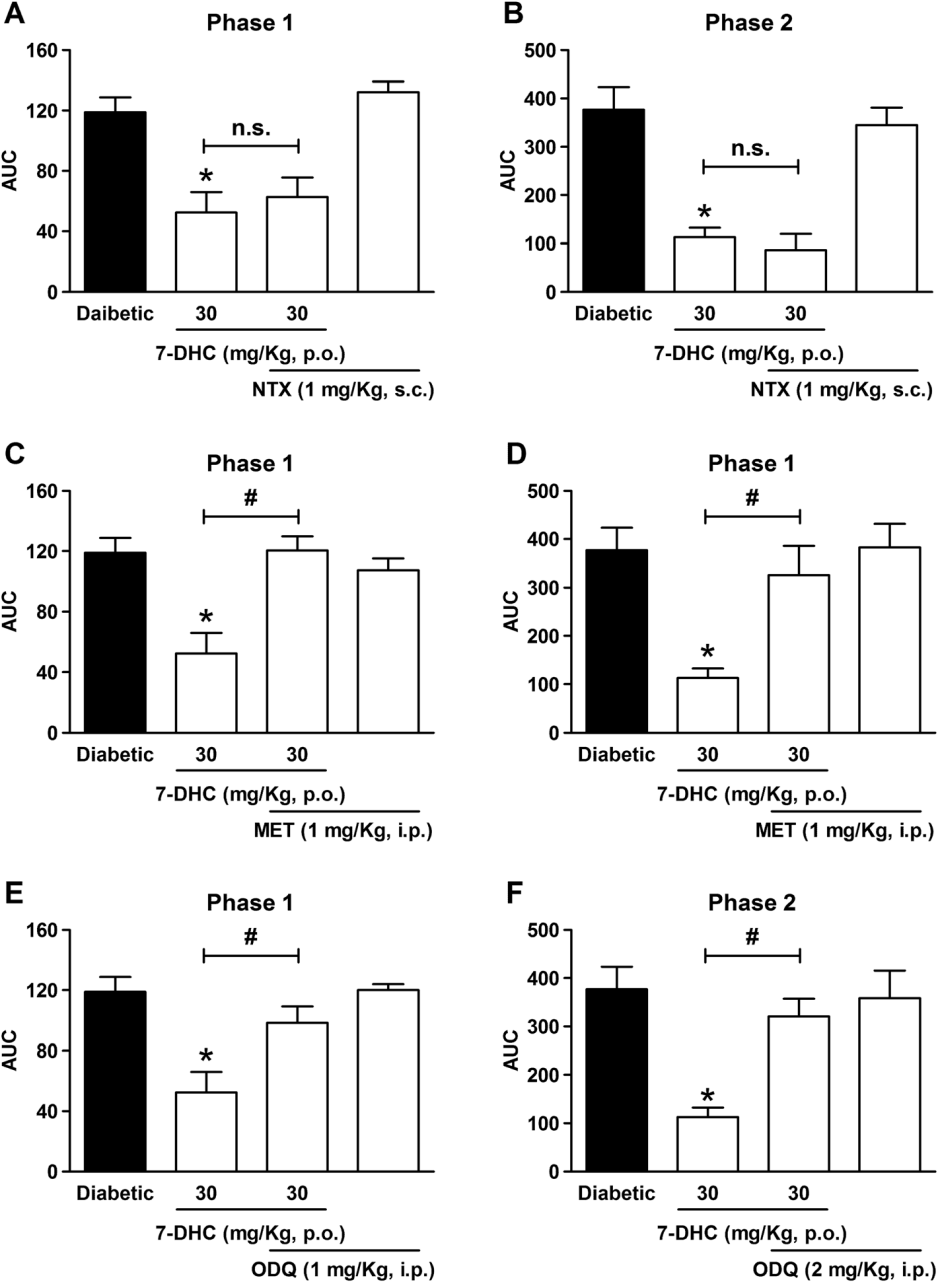
**Effect of systemic s.c. naltrexone (NTX, opioid antagonist, A-B), i.p. methiothepin (MET, 5-HT antagonist, C-D), and i.p. 1H-[1,2,4]oxadiazolo[4,3-a]quinoxalin-1-one (ODQ, guanylyl cyclase inhibitor, E-F) on p.o. 7-hydroxy-3,4-dihydrocadalin-induced (7-DHC) antihyperalgesic effect during both phases of 0.5% formalin test in diabetic rats (3 weeks).** 7-DHC was given 1 h before formalin injection whereas antagonists or inhibitor were given 30 min after oral 7-DHC administration. Antagonists or inhibitor did not show any nociceptive effect by themselves at the tested dose for its administration with 7-DHC. Data are expressed in bars as average of the area under the curve (AUC) ± E.E.M. of six animals per group. *Significantly different (*P* < 0.05) from control group (diabetic) and ^#^significantly different (*P* < 0.05) from 7-DHC group (30 mg/kg), as determined by one-way ANOVA followed by the Tukey’s test. AUC: Area under the curve; n.s.: not significant.

In order to assess the participation of 5-HT and opioid receptors as well as guanylyl cyclase enzyme on 7-hydroxy-3,4-dihydrocadalin-induced antihyperalgesic effect in the 1% formalin test, methiothepin (non-selective 5-HT receptor antagonist), naltrexone (non-selective opioid receptor antagonist) or ODQ (selective guanylyl cyclase inhibitor) were injected 30 min after oral administration of 7-hydroxy-3,4-dihydrocadalin. Figure 4 shows that subcutaneous administration of 1 mg/kg naltrexone (Figure 4A,B) was not able to block 7-hydroxy-3,4-dihydrocadalin-induced antihyperalgesic activity in any phase of formalin test (Figure 4A,B). In marked contrast, intraperitoneal administration of 1 mg/kg methiothepin (Figure 4C,D) or 2 mg/kg ODQ (Figure 4E,F) significantly prevented the antihyperalgesic effect of 7-hydroxy-3,4-dihydrocadalin in both phases of this test. The doses of naltrexone, methiothepin or ODQ did not modify per se formalin-induced nociceptive behavior and such doses were chosen on the basis of previously results obtained in the laboratory and in the literature [36,37].

### Antioxidative effect of acute administration of 7-hydroxy-3,4-dihydrocadalin in diabetic rats

**Figure 5:**
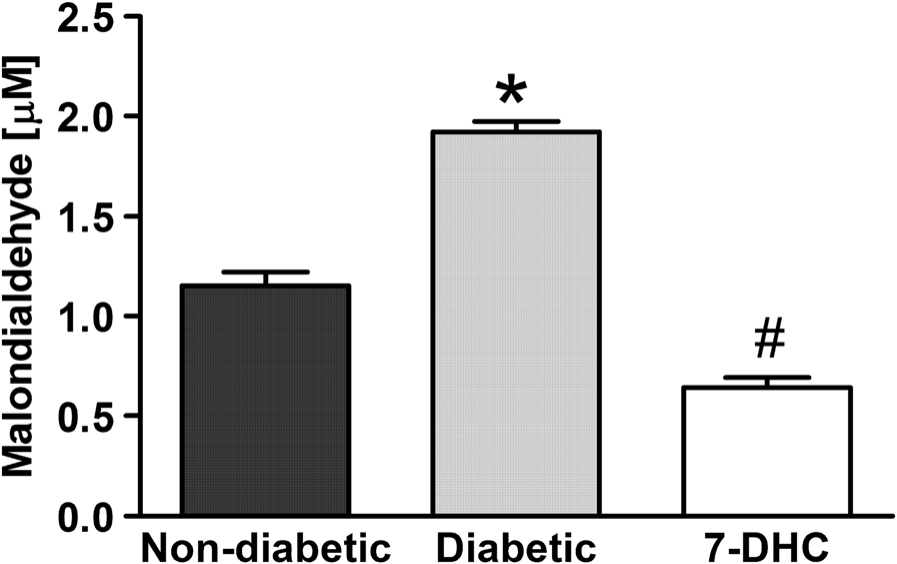
**Effect of acute (30 mg/kg) oral administration of 7-hydroxy-3,4-dihydrocadalin (7-DHC) on malondialdehyde concentration in plasma of diabetic rats submitted to 60 mg/kg streptozotocin 3 weeks before.** 7-DHC was given 1 h before blood sampling. Bars are the mean ± E.E.M. for 5 animals. *Significantly different from the non-diabetic group and ^#^significantly different (*P* < 0.05) from its respective control group (diabetic), as determined by one-way ANOVA followed by the Tukey’s test.

Three weeks after streptozotocin, but not distilled water, a significant increase of malondialdehyde concentration was observed in plasma samples. This increase was markedly (*P* < 0.05) reduced by oral acute treatment of 7-hydroxy-3,4-dihydrocadalin (30 mg/kg, -60 min) (Figure 5). In addition, 7-hydroxy-3,4-dihydrocadalin did not significantly modify serum malondialdehyde concentration in non-diabetic rats (data not shown).

### Effect of acute administration of 7-hydroxy-3,4-dihydrocadalin on motor activity of diabetic rats

**Figure 6:**
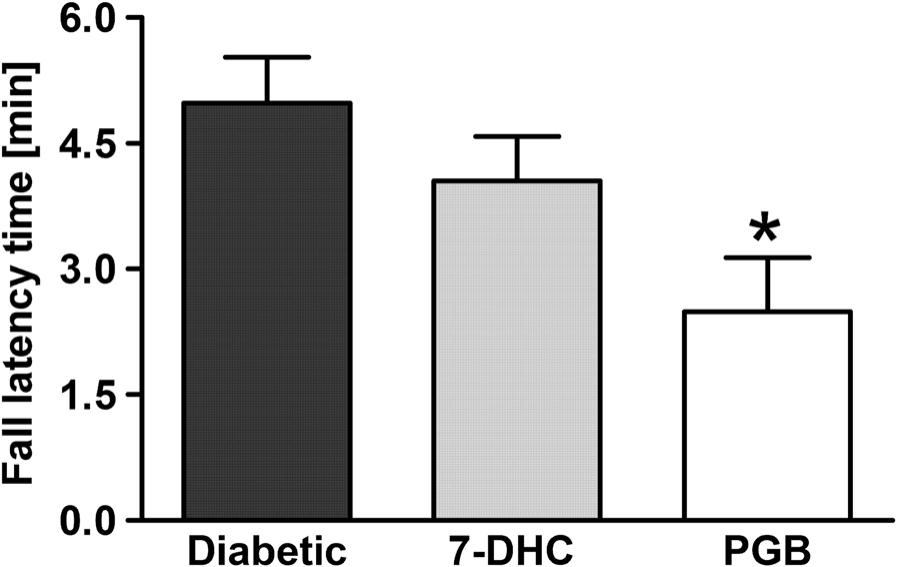
**Effect of oral acute 30 mg/Kg 7-hydroxy-3,4-dihydrocadalin (7-DHC) or 10 mg/kg pregabalin (PGB) administration on motor coordination of diabetic rats submitted to 60 mg/kg streptozotocin 3 weeks before.** 7-DHC and PGB were given 1 h before motor coordination testing. Bars are the mean ± E.E.M. for 5 animals. *Significantly different (*P* < 0.05) from control group (diabetic), as determined by one-way ANOVA followed by the Tukey’s test.

To determine motor side-effects of acute oral administration (-60 min) of vehicle or equieffective doses of 7-hydroxy-3,4-dihydrocadalin (30 mg/kg) and pregabalin (10 mg/kg) in diabetic rats (3 weeks) was performed the rota-rod test. Under these conditions, pregabalin, but not 7-hydroxy-3,4-dihydrocadalin, significantly (*P* < 0.05) reduced motor activity of streptozotocin-induced diabetic rats compared to vehicle group (Figure 6).

### Antiallodynic effect of acute administration of 7-hydroxy-3,4-dihydrocadalin in diabetic rats

**Figure 7:**
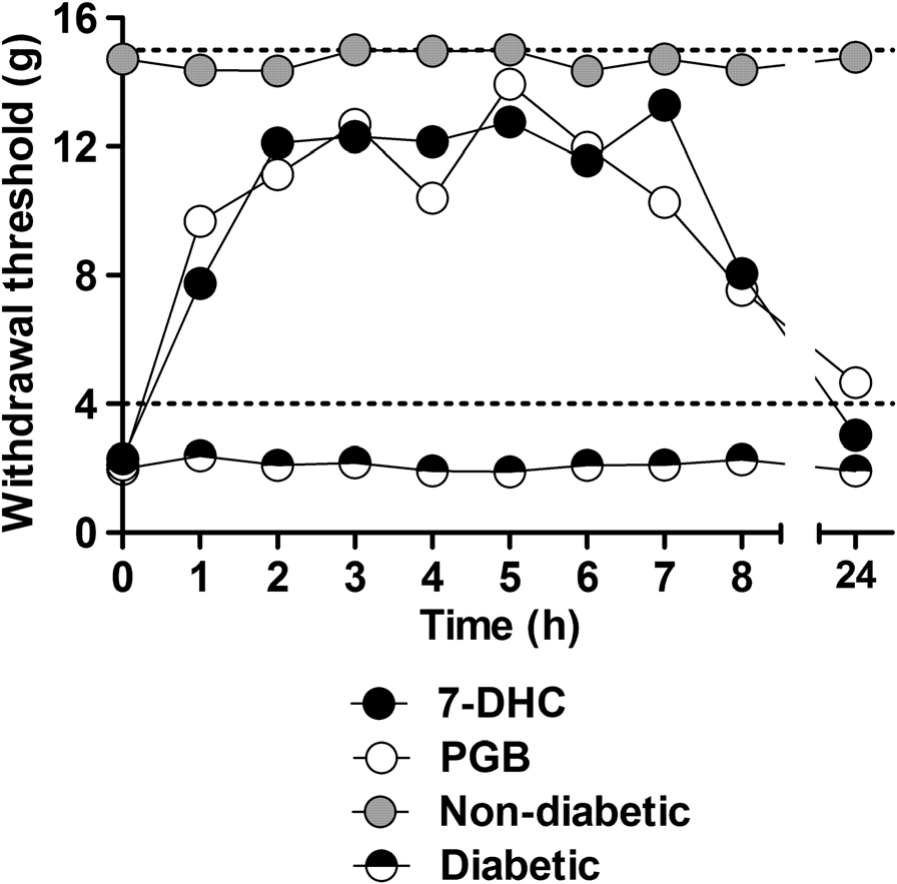
**Time course of the antiallodynic effect observed after acute oral of 30 mg/kg 7-hydroxy-3,4-dihydriocadalin (7-DHC) or 10 mg/kg pregabalin (PGB) administration in rats administered with 60 mg/kg streptozotocin (STZ) 6 weeks before experiment.** The non-diabetic group is placed as a reference of the maximum possible effect, whereas diabetic group shows that antiallodynic effect is induced by 7-DHC or PGB, but not by vehicle. Data are presented as mean ± E.E.M. for 6 animals. Both treatments were significantly different (*P* < 0.05) from control group (diabetic) in the range of 1 to 8 h, as was determined by two-way ANOVA followed by Bonferroni’s test, but asterisks were removed for the sake of clarity.

In order to confirm the antineuropathic effect of 7-hydroxy-3,4-dihydrocadalin, streptozotocin-diabetic rats were evaluated with the von Frey filaments. Hyperglycemic rats developed tactile allodynia around 6 weeks after streptozotocin injection. In marked contrast, non-diabetic animals remained with a normal threshold during the 6 weeks. No autotomy behavior was ever observed during the experiment. On this condition, oral acute administration of 7-hydroxy-3,4-dihydrocadalin (30 mg/kg) or pregabalin (10 mg/kg), but not vehicle, increased the withdrawal threshold in diabetic rats. Both drugs reached the maximal antiallodynic effect at 2 h and it lasted for 8 h at the tested doses, 24 h after oral administration the antiallodynic effect had disappeared (Figure 7). In a similar way to the formalin test, 7-hydroxy-3,4-dihydrocadalin (69.9 ± 4.0%) was equi-effective to pregabalin (67.8 ± 4.1%) in this model.

### Antihyperalgesic and antiallodynic effects of chronic administration of 7-hydroxy-3,4-dihydrocadalin in diabetic mice

**Figure 8:**
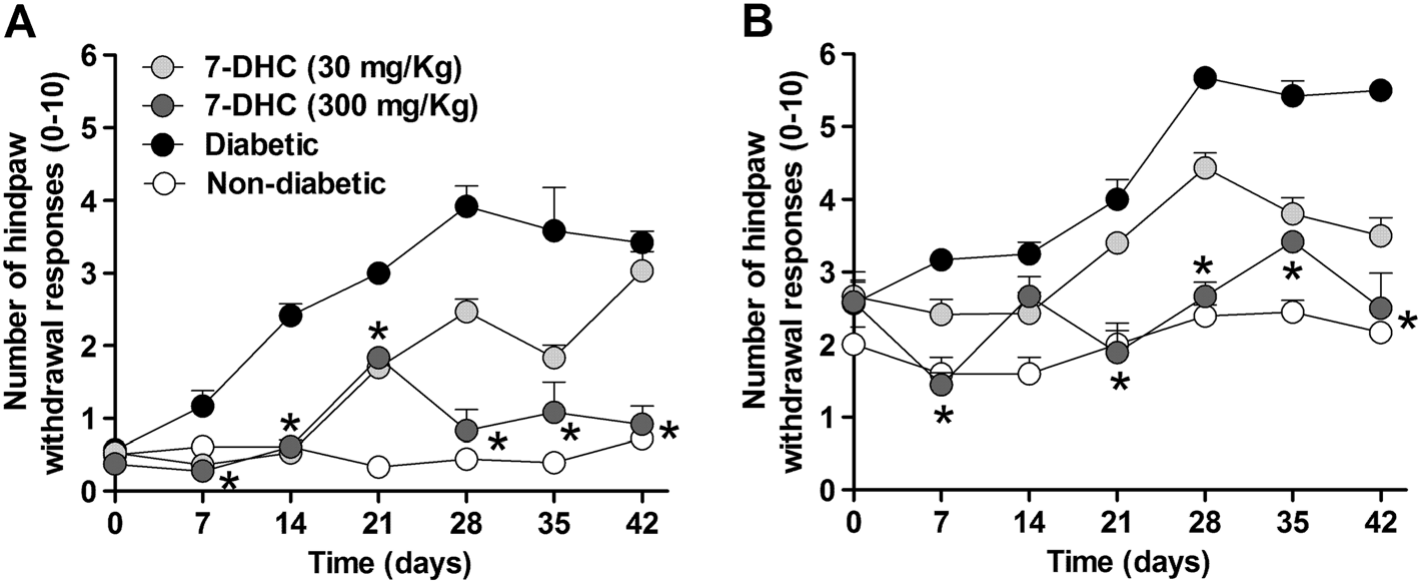
**Antineuropathic effect of chronic oral administration of 30 or 300 mg/kg of 7-hydroxy-3,4-dihydrocadalin (7-DHC), on Monday, Wednesday and Friday for 6 weeks, on 200 mg/kg streptozotocin-induced hyperalgesia and allodynia in diabetic mice.** Data are presented as mean ± E.E.M. for 6 animals. *Significantly different (*P* < 0.05) from control group (diabetic), as was determined by two-way ANOVA followed by Bonferroni’s test.

Streptozotocin (200 mg/kg), but not distilled water (0.1 mL/kg), injection caused hyperglycemia in mice. The ICR mice had 120.7 ± 9.4 and 126.6 ± 10.6 mg/dL of fed blood glucose levels before distilled water or streptozotocin injection, and 121.2 ± 6.5 and 551.2 ± 13.8 mg/dL 6 weeks after distilled water or streptozotocin injection, respectively. Furthermore, diabetic mice had an increase in mechanical hyperalgesia and allodynia from the first week compared to vehicle group. Oral administration of 7-hydroxy-3,4-dihydrocadalin, but not vehicle, on Mondays, Wednesday and Fridays during 6 weeks prevented in a dose-dependent manner (30–300 mg/kg) and significantly (*P* < 0.05) the development of mechanical hyperalgesia and allodynia in diabetic mice (Figure 8). Evaluations were done weekly (on Mondays), 72 h after the last oral administration of 7-hydroxy-3,4-dihydrocadalin or vehicle in these animals.

## Discussion

The current study was performed to determine the therapeutic potential of 7-hydroxy-3,4-dihydrocadalin in the treatment of streptozotocin-induced painful peripheral neuropathy in rodents. In our study, dose-response curve of 7-hydroxy-3,4-dihydrocadalin acute administration presented a dose-dependent antihyperalgesic effect in the formalin test in diabetic rats. In addition, this drug showed antiallodynic activity in the streptozotocin-induced tactile allodynia model. In both tests, the antineuropathic effect was equieffective to pregabalin, a drug of first line in the treatment of neuropathic pain [40,41]. In line with these results, chronic administration of oral 7-hydroxy-3,4-dihydrocadalin also prevented the development of mechanical hyperalgesia and allodynia in mice. In contrast with the doses used in rats, the antineuropathic activity was observed in mice with doses of 7-hydroxy-3,4-dihydrocadalin 10 times greater. Differences could be attributed to the specie used (rats versus mice) since it is well known that hepatic metabolism of mice is more accelerated than rats [42-44]. To the best of our knowledge, this is the first report about the antineuropathic activity of 7-hydroxy-3,4-dihydrocadalin. Our study agree with a previous study showing the antinociceptive effect of 7-hydroxy-3,4-dihydrocadalin in non-diabetic rats submitted to 1% formalin test and Randall-Selitto model [18]. Taken together, data suggest that 7-hydroxy-3,4-dihydrocadalin exerts antinociceptive activity in neuropathic and inflammatory pain models. In addition, results seem to point out that 7-hydroxy-3,4-dihydrocadalin has an advantage on the rest of antineuropathic drugs used in the clinic to treat painful diabetic neuropathy as this compound not only reduces neuropathic pain intensity, but also prevents the development of this condition. In this regard, results indicate that 7-hydroxy-3,4-dihydrocadalin produced its antinociceptive effect by a glucose levels-independent mechanism as this compound was not able to modify blood glucose in an acute or chronic administration schedule.

The mechanism of the antinociceptive effect of 7-hydroxy-3,4-dihydrocadalin in neuropathic diabetic rats is unknown. However, considering that opioid, serotonergic and nitrergic systems are the most promising targets for the treatment of peripheral diabetic neuropathy or neuropathic pain by natural products [9-13], and it has been reported that 7-hydroxy-3,4-dihydrocadalin activates 5-HT_1_, but not opioid receptors [18], we decided to test if these mechanisms participated in the antineuropathic activity of 7-hydroxy-3,4-dihydrocadalin in diabetic rats. In this regard, we found that the non-selective 5-HT receptor antagonist methiothepin [45] was able to prevent 7-hydroxy-3,4-dihydrocadalin-induced antihyperalgesia in three-week diabetic rats. This result suggests that some of the 5-HT receptors may play a role in the antihyperalgesic effect of 7-hydroxy-3,4-dihydrocadalin in streptozotocin-induced diabetic rats. In support of this, there is evidence that activation of 5-HT_1A/B/D_ receptors participate in the peripheral antinociceptive effect observed with 7-hydroxy-3,4-dihydrocadalin in formalin-induced inflammatory pain [18]. The participation of 5-HT_1A_ seems unlikely because the most of literature points out a pronociceptive role of 5-HT_1A_ receptor in several models of neuropathic pain [46-48] including thermal hyperalgesia in streptozotocin-induced diabetic mice [49], notwithstanding, this has been disputed [50,51]. On the other hand, evidence of 5-HT_1B/D_ receptors in neuropathic pain suggests that they are linked to antinociception [52] and could explain the antihyperalgesic effect of 7-hydroxy-3,4-dihydrocadalin.

In the current study we also found that ODQ prevented 7-hydroxy-3,4-dihydrocadalin -induced antihyperalgesic effect in the formalin test. Since ODQ is a guanylyl cyclase inhibitor [53], our data suggest that antihyperalgesia observed with 7-hydroxy-3,4-dihydrocadalin in diabetic rats could be due to activation of guanylyl cyclase. Hypothesizing, this activation would lead to the synthesis of cyclic GMP, which in turn, would activate the protein kinase G and finally it could open potassium channels to hyperpolarize the nociceptive neurons, as with the natural products that activate this enzyme [12,13].

On marked contrast, subcutaneous administration of the non-selective opioid receptor antagonist naltrexone did not prevent the antihyperalagesic effect induced by the acute administration of 7-hydroxy-3,4-dihydrocadalin. This result indicates that opioid receptors do not play a role in the antihyperalgesic effect of 7-hydroxy-3,4-dihydrocadalin in streptozotocin-induced diabetic rats of 3 weeks. The lack of effect of naltrexone is not due to the dose used in this work, since this dose has been reported to be effective in previous studies in diabetic rats with similar characteristics [30,54]. Taken together, results suggest that 7-hydroxy-3,4-dihydrocadalin exerts its antihyperalgesic affect through activation of 5-HT_1_ receptors and guanylyl cyclase enzyme, but not opioid receptors.

Additionally to the described mechanisms, 7-hydroxy-3,4-dihydrocadalin decreased the malondialdehyde concentration in diabetic rats to baseline values in non-diabetic rats. Our data agree with evidence demonstrating a potent scavenging activity of 7-hydroxy-3,4-dihydrocadalin on diphenyl-p-picrylhydrazyl radical or microsomal and mitocondrial lipid peroxidation [19-21]. Furthermore, 7-hydroxy-3,4-dihydrocadalin has shown to be effective to protect red cells against oxidative haemolysis [20] or mitochondrial enzyme activity against oxidative stress [21]. Antioxidant activity of 7-hydroxy-3,4-dihydrocadalin results of interest as pathogenesis of diabetic neuropathy is related with metabolic changes in several pathways that interact in a mutually facilitatory fashion to cause an imbalance in the mitochondrial redox state and an increment in the expression of pro-inflammatory genes producing neuronal hypersensitivity [3,5]. Remarkably, 7-hydroxy-3,4-dihydrocadalin has also demonstrated anti-inflammatory properties on the croton oil-induced edema test in mouse ear [22] and carrageenan-induced paw edema model [18]. Speculating, 7-hydroxy-3,4-dihydrocadalin could be useful to treat the diabetic neuropathy in a more integrative manner as it combines antineuropathic, antioxidant and possibly anti-inflammatory properties.

Although pregabalin is clinically considered as the gold standard for the treatment of painful diabetic neuropathy in human beings [55], its use is limited due to side effects such as sedation, dizziness and somnolence that affects motor coordination [56,57]. For this reason, equi-effective doses of 7-hydroxy-3,4-dihydrocadalin and pregabalin were compared in the rota-rod test. Interestingly, pregabalin, but not 7-hydroxy-3,4-dihydrocadalin, affected motor coordination in diabetic rats. Our results seem to point out that notwithstanding the similar antineuropathic activity between both drugs, 7-hydroxy-3,4-dihydrocadalin might present an advantage on pregabalin in the diabetic neuropathy treatment since 7-hydroxy-3,4-dihydrocadalin did not produce motor side-effects. However, further studies are necessary to assess the safety of oral acute and chronic administration of 7-hydroxy-3,4-dihydrocadalin. In addition, a clinical study of efficacy and safety study in patients with painful diabetic neuropathy is required to evaluate the real therapeutic potential of this compound in the diabetic neuropathy.

## Conclusions

Data suggest that 7-hydroxy-3,4-dihydrocadalin, an active compound of Mexican arnica with antioxidant, anti-inflammatory and antinociceptive activities, has therapeutic potential for diabetic neuropathy treatment, since oral administration of this compound decreased hyperalgesia and allodynia in a similar way than pregabalin in rats without affecting motor coordination. In addition, this drug may be useful to prevent the development of painful diabetic neuropathy as its chronic administration avoided the progress of neuropathic pain in mice. The antinociceptive effect seems to involve activation of 5-HT_1_ receptors and guanylyl cyclase enzyme, whereas its neuroprotective mechanisms could be more related to the antioxidant properties of 7-hydroxy-3,4-dihydrocadalin.

## Competing interests

The authors declare that they have no competing interests.

## Authors’ contributions

All authors have read and approved the final manuscript. HIRG, VGS and AN designed, performed, and supervised the experiments, analyzed the data, prepared the figures and wrote the manuscript. MRA and JCHC performed the experiments. GRG and JETL coordinated the project, helped to interpret the data and edited the manuscript.

## Authors’ information

This work was part of the B.Sc. dissertation of Magali Ramírez-Aguilar. Héctor Isaac Rocha-González was on a DGAPA-UNAM postdoctoral fellowship.

## Pre-publication history

The pre-publication history for this paper can be accessed here:

http://www.biomedcentral.com/1472-6882/14/129/prepub
